# Transcontinental Movement of Asian Genotype Chikungunya Virus

**DOI:** 10.3201/eid2008.140268

**Published:** 2014-08

**Authors:** Robert S. Lanciotti, Anne Marie Valadere

**Affiliations:** Centers for Disease Control and Prevention, Fort Collins, Colorado, USA (R.S. Lanciotti);; Caribbean Public Health Agency, Port of Spain, Trinidad and Tobago (A.M. Valadere)

**Keywords:** chikungunya virus, CHIKV, Asian genotype, mosquito transmitted virus, Togaviridae, Alphavirus, epidemics, polyarthralgia, chikungunya fever, viruses, Southeast Asia, the Caribbean, British Virgin Islands, Yap, China, the Philippines

**To the Editor:** Chikungunya virus (CHIKV), a mosquito–transmitted virus (family *Togaviridae,* genus *Alphavirus*), was first isolated >60 years ago in Africa and is responsible for epidemics of acute polyarthralgia. During CHIKV epidemics, the transmission cycle is from humans to mosquitoes, with no intervening amplifying host, and the virus can rapidly disseminate, infecting large numbers of persons. Epidemics have been described in Africa, the Middle East, Europe, India, and Southeast Asia. On the basis of detailed clinical descriptions of the disease, chikungunya fever, it appears that CHIKV caused epidemics in the Caribbean (St. Thomas, US Virgin Islands) and the southeastern coastal United States during the early 19th century (*1*).

Genetic studies show that the virus has evolved into 3 distinct genotypes: West African, East/Central/South African (ECSA), and Asian (*2*). The genotypes likely indicate independent evolution of the virus in historically isolated areas. Phenotypic differences have been described between genotypes and between individual strains, most notably an E1 mutation among some ECSA strains, which facilitates replication in *Aedes albopictus* mosquitoes (*3*). However, more recently, the movement of virus genotypes has increased dramatically, probably as a direct result of increased movement of humans and increased commercial trade. Beginning in 2005 and through 2006, the ECSA genotype virus was responsible for an explosive epidemic, during which the virus moved from coastal Kenya to islands adjacent to southeastern Africa and then to India, where >1 million cases were recorded (*2*). During this time, imported cases were reported worldwide, and in some instances, autochthonous transmission was detected in distal locations (*4*,*5*).

In October 2013, the arbovirus diagnostic laboratory at the Centers for Disease Control and Prevention (CDC; Fort Collins, CO, USA) detected CHIKV in human serum specimens from Yap State, Federated States of Micronesia; the specimens were collected during an epidemic of disease clinically compatible with chikungunya fever. In December 2013, the French National Reference Centre for arboviruses confirmed that CHIKV was responsible for an epidemic occurring on St. Martin Island, French West Indies, in the Caribbean (*6*). In January 2014, the Caribbean Public Health Agency detected CHIKV in 2 human serum specimens from the British Virgin Islands (BVI); the samples were subsequently confirmed by CDC to be positive for CHIKV.

By using next-generation sequencing, we determined the complete nucleotide sequence for 1 of the CHIKV specimens detected in BVI and for 2 of the CHIKV specimens detected in Yap. DNA libraries for next-generation sequencing were prepared directly from RNA extracted from serum, and the amplified libraries were sequenced by using the Ion Torrent Personal Genome Machine (Life Technologies, Grand Island, NY, USA). The CLC Genomics Workbench (CLC bio, Aarhus, Denmark) and Lasergene NextGen (DNASTAR, Madison, WI, USA) were used to analyze and assemble raw sequence reads. ClustalW (www.ebi.ac.uk/Tools/msa/clustalw2/) was used to align the complete genome sequences with a variety of CHIKV sequences, representing the 3 genotypes, from GenBank. Nearly identical phylogenetic trees were generated by several methods (i.e., minimum evolution, maximum likelihood, neighbor joining); a representative neighbor-joining tree generated and analyzed with 1,000 replicates for bootstrap testing is shown in the Figure.

**Figure:**
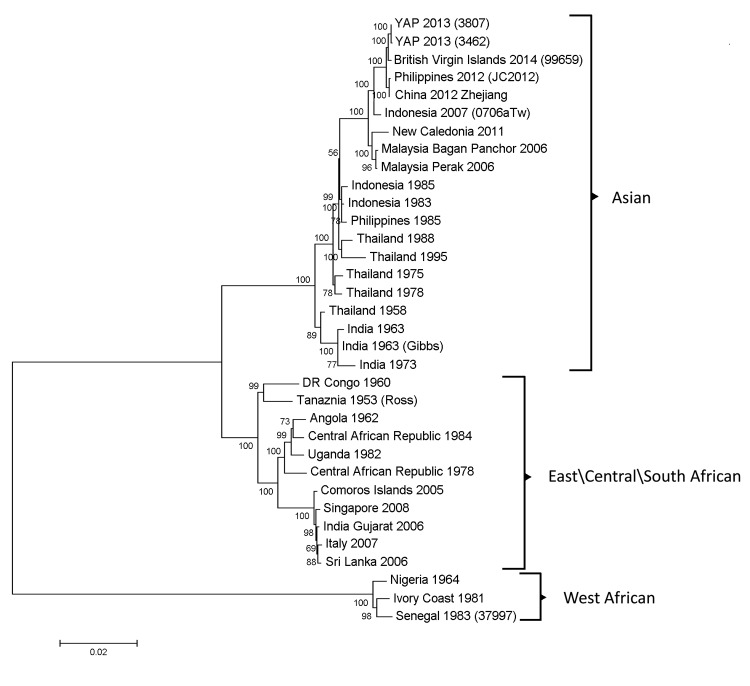
Phylogenetic tree derived by neighbor-joining methods (1,000 bootstrap replications) using complete genome sequences of chikungunya viruses obtained from GenBank. Scale bar represents the number of nucleotide substitutions per site. Genotypes are indicated on the right. GenBank accession numbers for viruses used for construction of the tree follow: Yap 2013–3807 (KJ451622), Yap 2013–3462 (KJ451623), British Virgin Islands-99659 (KJ451624), Philippines 2012-JC2012 (KC488650), China 2012 Zhejiang (KF318729), Indonesia 2007–0706aTw (FJ807897), New Caledonia 2011 (HE806461), Malaysia 2006 Bagan Panchor (EU703759), Malaysia 2006 Perak (EU703760), Indonesia 1985 (HM045797), Indonesia 1983 (HM045791), Philippines 1985 (HM045790), Thailand 1988 (HM045789), Thailand 1995 (HM045796),Thailand 1975 (HM045814),Thailand 1978 (HM045808),Thailand 1958 (HM045810), India 1963 (HM045803), India 1963 Gibbs (HM045813), India 1973 (HM045788), DR Congo 1960 (HM045809), Tanzania 1953 Ross (AF490259), Angola 1962 (HM045823), Central African Republic 1984 (HM045784), Uganda 1982 (HM045812),Central African Republic 1978 (HM045822), Comoros 2005 (HQ456251), Singapore 2008 (FJ445510), India 2006 Gujarat (JF274082), Italy 2007 (EU244823), Sri Lanka 2006 (GU189061), Nigeria 1964 (HM045786), Ivory Coast 1981 (HM045818), and Senegal 1983–37997 (AY726732).

In agreement with findings in a recent report characterizing the 2013 CHIKV detected on St. Martin Island (*6*), the phylogenetic tree generated from our sequence data showed that the 2014 CHIKV from BVI is within the Asian genotype and is closely related to strains recently isolated in China and the Philippines. This finding supports the idea that a single CHIKV strain of the Asian genotype was recently introduced into the Caribbean and is currently moving throughout the region. The 2 CHIKVs isolated in Yap in 2013 are most closely related to the CHIKV from BVI, differing by only 18–19 nt.

The tree also demonstrates that the CHIKV strains from Yap, BVI, China, and the Philippines form a strongly supported clade (bootstrap of 1,000) within the Asian genotype (Figure). Within this clade, the CHIKVs detected in 2012 in Zhejiang Province, China, and the Philippines are nearly identical, differing by only 4 nt in the entire genome. However, there is some ambiguity regarding the exact origins of these 2 strains. The 2012 CHIKV from the Philippines is described in GenBank (www.ncbi.nlm.nih.gov/nuccore/KC352904.1) as an “imported Chikungunya fever case in Ningbo port”; the virus was isolated and identified in Ningbo, China, but was detected in samples from a traveler from the Philippines (GenBank accession nos. KC352904.1 and KC488650.1). The 2012 Zhejiang CHIKV was detected and characterized in Zhejiang Province, but the virus was from a sailor who traveled around Southeast Asia; therefore, the exact origin of this virus is also unknown (*7*).

The striking similarity between the 2012 CHIKVs from the Philippines and Zhejiang Province suggests a common origin, perhaps the Philippines, where CHIKV transmission was documented in 2012 and 2013. Regardless of the exact origins of these 2 strains, it is clear that the CHIKV strain currently moving throughout the Caribbean originated from a CHIKV strain that was recently circulating between China, the Philippines, and Yap in Southeast Asia.
